# The self as destination or illusion: a comparative study of individuation in Jung and self-transcendence in Buddhist Vijñānavāda

**DOI:** 10.3389/fpsyg.2026.1858440

**Published:** 2026-06-30

**Authors:** Yiwen Zhang

**Affiliations:** Department of Philosophy, School of Humanities, Tsinghua University, Beijing, China

**Keywords:** ālaya-vijñāna, comparative psychology, individuation, manas, self archetype, turning consciousness into wisdom, Vijñānavāda

## Abstract

**Introduction:**

This article examines whether Jung’s theory of individuation reaches a structural limit that Vijnanvada (Yogacara) Buddhist philosophy can identify and continue. Prior comparative scholarship has mapped Jungian archetypes onto Buddhist categories; this study argues that such approaches conceal a more fundamental asymmetry between the two traditions.

**Methods:**

The study employs structural-comparative analysis of Jung’s Collected Works (vols 9/1, 9/2, 10, 11, 12) and the Cheng Weishi Lun (CBETA T31, no. 1585), with secondary scholarship in Buddhist studies, Jungian psychology, and phenomenology. The comparison proceeds at the level of cognitive architecture and is applied reflexively to both traditions.

**Results:**

Four findings are established. (1) Both traditions posit a subliminal mind (collective unconscious / alaya-vijnana) in response to the insufficiency of surface consciousness. (2) Jung’s integrative methodology misreads the structural self-grasping of manas as content available for integration. (3) The Jungian Self archetype reproduces at a more sophisticated level the same atma-graha structure that manas enacts in lived experience. (4) The Vijnanvada doctrine of ‘turning consciousness into wisdom’ (zhuan shi cheng zhi) articulates a transformation of cognitive mode from self-assumption to samata-jnana that Jung’s framework approaches but does not formulate.

**Discussion:**

Individuation arrives at the threshold of cognitive transformation without articulating the means to cross it. Vijnanvada offers a possible continuation: individuation functions as precondition for the subtler work of transforming the structural orientation of cognition itself. Both frameworks are subject to reciprocal epistemological constraints and reflexive hermeneutic suspicion.

## 1 Introduction

The relationship between Jungian psychology and Buddhist thought has generated substantial comparative scholarship since the mid-twentieth century. Jung himself was a persistent, if selective, reader of Eastern materials. His forewords to Suzuki Daisetsu’s Introduction to Zen Buddhism, his psychological commentaries on The Tibetan Book of the Dead and The Secret of the Golden Flower, and his sustained engagement with mandala imagery all testify to an engagement with Asian traditions that was, however, consistently governed by the prior commitments of his own theoretical system. As Heisig (1999, p. 94) demonstrated in a systematic study, Jung assimilated Eastern materials to the logic of archetypes and individuation rather than encountering them on their own terms: “nowhere in his study of myth, primal religions, or Eastern thought does Jung seriously entertain the idea that his archetype of the Self may be based more on a monotheistic preconception than on the actual data.” Clarke (1994, p. 78) similarly observed that despite Jung’s frequent recourse to Eastern symbolism, the spiritual nourishment he drew personally from Christian imagery likely exceeded what he derived from any Asian tradition. These observations identify a productive starting point: the relationship between Jung and Buddhism is not one of influence or borrowing but of structural tension between two independent accounts of the deep architecture of mind.^1^

These observations should be situated within a broader history of encounter between Western psychology and Buddhist thought. The Freud-Jung exchange on religion set the initial terms: where Freud (1927/1961) treated religion as a wish-fulfilling illusion rooted in infantile helplessness, Jung saw in it an authentic expression of the collective unconscious (Segall, 2023). The first sustained engagement between psychoanalysis and Buddhism came through Alexander (1931), who interpreted nirvāṇa as regression to the womb and meditative absorptions as stages of self-induced melancholia—an interpretive framework that, however reductive, opened the door to more sophisticated encounters. Fromm et al. (1960) attempted a substantive dialogue between Zen and psychoanalysis, arguing that both traditions addressed the alienation of modern consciousness, though their conversation remained at the level of therapeutic aspiration rather than structural comparison. Moacanin (1986) pursued the comparison more systematically in her study of Jung’s psychology and Tibetan Buddhism, identifying significant structural parallels between the individuation process and the stages of tantric transformation, while noting that the two systems presuppose fundamentally different ontologies. More recently, a generation of Buddhist practitioner-psychoanalysts—including Barry Magid, Mark Epstein, and the Jungian analyst Polly Young-Eisendrath—have worked to integrate Buddhist insight into clinical practice, typically by “naturalizing” Buddhism in ways that downplay its soteriological and metaphysical commitments (Segall, 2023). Against this background, the present article’s contribution lies not in proposing another rapprochement between the two traditions but in pressing the comparison to a deeper structural level: the question of whether the cognitive mode in which individuation operates is itself susceptible to transformation.

Coward (1985) provided an earlier comparative framework in his edited volume on Jung and Eastern thought, bringing together scholars from both traditions to examine the points of contact and divergence. His contribution was to show that the comparison could not be conducted at the level of isolated concepts—archetype versus dharma, Self versus ātman—but required attention to the structural logics within which these concepts function. The present article follows Coward’s methodological insight while pushing the comparison further than he was able to do, into the territory of cognitive structure and its possible transformation. It is worth noting that the transpersonal psychology movement, associated with Stanislav Grof, Ken Wilber, and others, also attempted to bridge Western psychology and Eastern contemplative traditions, but its theoretical foundations have been questioned for a tendency toward syncretic generalization that obscures the specific mechanisms at work in each tradition (Segall, 2020, pp. 78–113). The present article avoids syncretic claims and confines itself to structural comparison.

This article selects Vijñānavāda—the Yogācāra school of Mahāyāna Buddhism—as the Buddhist interlocutor for reasons that are theoretically specific. Among Buddhist philosophical schools, Vijñānavāda offers the most technically developed analysis of the structure of mind: its eight-consciousness model, its theory of karmic seeds and habitual impressions (bīja and vāsanā), and its account of cognitive transformation called “turning consciousness into wisdom” (zhuǎn shí chéng zhì, 轉識成智) constitute a framework of exceptional analytical precision. Waldron (2003a) established that the ālaya-vijñāna concept emerged precisely as a response to theoretical gaps exposed by Abhidharma analysis of consciousness—gaps structurally analogous to those that drove Jung beyond Freud’s model of the unconscious. Schmithausen (1987) documented the concept’s philological and philosophical development with a rigor that places it beyond reasonable scholarly dispute. Nī (2002) has demonstrated, through his comparison of Vijñānavāda with Husserlian phenomenology, that the two traditions share sufficient methodological common ground to sustain structural comparison without reducing either to the categories of the other.

The central thesis of this article is that Jung’s explicit theoretical model of individuation reaches the boundary of what it can articulate with its available conceptual resources, and that it stops at the figure of the Self archetype. The Vijñānavāda eight-consciousness framework, the tripartite structure of cognition, the karmic-seed theory, and the doctrine of cognitive transformation constitute resources that Jung’s model gestures toward but does not generate from within its own theoretical horizon. The argument is not that Vijñānavāda is superior to Jung—the epistemological limits of both traditions are addressed in Section 6—but that where Jung’s analysis stops, Vijñānavāda offers not a replacement but a possible continuation. Three qualifications govern this thesis throughout. First, the claim concerns the architecture of Jung’s published theoretical model, not the phenomenology of individuation as lived in the consulting room; no clinical evidence for a developmental ceiling in actual analysands is asserted here, and none could be derived from the textual analysis this article offers. Second, the claim is advanced in full awareness that the later Jung, above all in Mysterium Coniunctionis, pressed toward a more apophatic and less centered understanding of the Self (Jung, 1963), a development examined directly in Section 5. Third, the standpoint from which the comparison is conducted is itself placed under examination: Section 6 applies to Vijñānavāda’s self-description the same hermeneutics of suspicion that the article applies to Jung.

## 2 Methods

This study employs structural-comparative analysis as its primary method. Rather than mapping surface-level conceptual correspondences between Jungian and Vijñānavāda terminology, the analysis proceeds at the level of cognitive architecture: it examines how each tradition accounts for the structure of subliminal mind, the operations of self-grasping, and the conditions under which cognitive transformation is possible.

Primary sources consulted include Jung’s Collected Works (volumes 9/1, 9/2, 10, 11, and 12) and the Cheng Weishi Lun (T31, no. 1585, CBETA digital edition), read in the original Chinese with reference to Cook (1999) English translation and Lin’s (2000) commentary. All quotations from the Cheng Weishi Lun are translated from the Chinese by the author.

Secondary scholarship in Buddhist studies, Jungian psychology, and phenomenology was selected for methodological rigor and disciplinary diversity, with preference given to sources that engage primary texts directly rather than relying on received interpretive traditions. The comparative framework was developed inductively from the primary sources rather than imposed in advance, allowing structural asymmetries between the two traditions to emerge from the analysis itself.

Two methodological constraints deserve explicit statement. First, the analysis operates at the level of theoretical reconstruction. Its claims concern the conceptual resources and structural commitments of the two frameworks as articulated in their texts, not the clinical or contemplative outcomes those frameworks produce in practice; statements about what individuation does or does not accomplish are accordingly to be read as statements about Jung’s explicit model. Second, the comparison is conducted reflexively. Because the analysis draws on Vijñānavāda categories to diagnose a limit in Jung’s framework, it incurs an obligation to subject those categories to the same critical scrutiny; Section 6 discharges this obligation by examining whether the eight-consciousness model is itself a culturally conditioned symbolic system. Recent scholarship in analytical psychology (Colman, 2008; Roesler, 2012; Giegerich, 2012; Mills, 2013) and in the psychology of meditation (Vago and Silbersweig, 2012; Josipovic, 2014; Dahl et al., 2015) is engaged at the points where it bears on these two constraints.

## 3 The insufficiency of consciousness: the unconscious and Ālaya-vijñāna—two discoveries of a deeper repository

Jung’s theory of the unconscious developed through, and ultimately beyond, the framework he had inherited from Freud. Freud’s psychoanalysis had opened the domain of the personal unconscious—the repository of repressed memories, wishes, and traumas—to systematic investigation; Jung accepted this framework but found it insufficient in the face of his clinical material. Patients’ dreams and active imagination produced image-patterns with no discernible relation to personal history yet recurring across individuals with striking regularity, corresponding to motifs found in the mythological and religious traditions of culturally remote peoples and periods. From this evidence, Jung developed the hypothesis of the “collective unconscious”: beneath the personal unconscious shaped by individual experience lay a deeper layer common to all human beings, which he described as “a common substratum transcending all differences in culture and consciousness” (Jung, 1968, para. 11). He called the inhabitants of this deeper layer archetypes—formal possibilities for psychic organization, present at birth, not invented but only discovered. Jung also referred to this level of the psyche as “objective and impersonal” to distinguish it from the subjectivity of ego-consciousness: it was as if each individual were fitted out at birth with a built-in tradition whose survival and evolution were relatively independent of subsequent personal experience (Heisig, 1999, p. 80). Heisig (1999, p. 81) notes that Jung’s path to this conclusion was characteristically speculative: “like so many creative ideas, he had leaped to the conclusion and worked out later the logical route that would justify it as a consequence.”^2^

The concept of the collective unconscious, once formulated, served as the conceptual axis around which Jung reorganized his entire psychology. It provided the basis for his theory of archetypes, his account of the religious function of the psyche, and his teleological understanding of the individuation process. Unlike Freud, who saw the unconscious primarily as a repository of repressed material—material that had once been conscious but had been pushed below the threshold of awareness—Jung insisted that the collective unconscious was never conscious to begin with. Its contents were not repressed but simply not yet known, awaiting discovery through the symbolic encounter between ego and archetype. This distinction is significant because it means that the Jungian unconscious, at its deepest level, is not something to be recovered but something to be met for the first time. Ellenberger (1970) situated this distinction within the broader history of dynamic psychiatry, showing how Jung’s reconceptualization of the unconscious departed from the Freudian model in ways that aligned his psychology more closely with the Romantic tradition of Schelling and Carus than with the mechanistic tradition of Helmholtz and Brücke.

The Vijñānavāda development of a subliminal-mind theory responded to a different crisis but one structurally analogous. The early Buddhist six-consciousness model distributed cognition among eye-, ear-, nose-, tongue-, body-, and mental-consciousness. This framework provided no account of psychological continuity across interruptions of surface awareness: deep sleep, unconscious states, and the meditative absorption called nirodha-samāpatti, in which all mental activity is temporarily suspended. If mind consists only of these six discontinuous streams, what sustains karmic potential across such interruptions? What binds the conditions for rebirth when surface consciousness lapses? Yinshun (1992, pp. 30–32) traced how this theoretical pressure, building within the Abhidharma tradition’s own analytical momentum, gradually drove Buddhist thinkers toward the postulation of a deeper, continuous layer of mind.^3^ Schmithausen (1987, p. 12) documents that the concept of ālaya-vijñāna was introduced to address this problem, with its earliest appearance likely in a stratum of the Yogācārabhūmi that postulates a basal consciousness persisting in the bodily sense-faculties even when all other cognitive processes cease. As the Saṃdhinirmocana Sūtra subsequently elaborated, this consciousness is continuous and uninterrupted, concealed yet containing all phenomena, serving as the deepest ground on which all subsequent mental events arise. Xiong (1985, p. 115) provides a systematic account of the eleven names given to the eighth consciousness in the tradition, each foregrounding a different aspect of its function.

The structural parallel between the collective unconscious and ālaya-vijñāna is genuine: both are posited as solutions to the problem of accounting for psychic depths that surface consciousness cannot access or explain. Yet the differences in dynamic architecture are equally significant. Jung conceived the collective unconscious as a relatively stable repository—archetypes evolved “very slowly,” and Jung had no difficulty assuming them to be identical “for a contemporary Tokyo fishmonger and an ancient Egyptian pharaoh” (Heisig, 1999, p. 80). This stability is the condition for Jung’s comparative method: only because the collective unconscious is structurally constant across time and culture can cross-cultural comparison of mythological and symbolic materials reveal its architecture. Two caveats must immediately qualify this characterization. Slow evolution is not changelessness: Jung allows for the modification of archetypal forms across historical epochs (Jung, 1969), so the collective unconscious is stable relative to the timescale of an individual life rather than absolutely static. Contemporary analytical psychology has pressed this point further: Roesler (2012) reviews the evidence that archetypal patterns may be transmitted culturally rather than biologically, a reading under which they would be historically mutable in principle. Nor is the collective unconscious inert: Jung’s own governing metaphors of the unconscious as sea and matrix (Jung, 1968, para. 281) present a generative ground rather than a fixed deposit. The contrast developed below is therefore one of degree, timescale, and mechanism of change, not a dichotomy of the static against the dynamic.

The ālaya-vijñāna is, by contrast, constitutively dynamic. Waldron (2003a, pp. 89–92) has characterized the relation between ālaya-vijñāna and the six manifest consciousnesses as an “intrapsychic feedback process”: manifest cognitive events continuously imprint impressions (vāsanā) into ālaya-consciousness in the form of seeds (bīja), which in turn condition the arising of further cognitive events, which deposit further impressions, in an unbroken causal circuit. The Cheng Weishi Lun [Xuanzang (trans.), 2002, juan 1] states: “each sentient being possesses a fundamental consciousness that maintains its own continuity, carrying seeds through a unitary stream; this consciousness and all phenomena are mutually each other’s causes, through the power of habitual impressions.” Germano and Waldron (2006, p. 43) describe this mutual conditioning through the metaphor of waves on a stream: the underlying current of ālaya-consciousness supports the surface waves of perceptual consciousness by mediating embodied cognitive schemas and “holding” the causal potentials for future waves, while the surface waves incessantly infuse further seeds into the underlying stream. Each wave that arises is simultaneously a product of and a transformation of the stream itself. The text explicitly warns against reifying this dynamic: although ālaya-vijñāna arises continuously in a stream of instants, “it is not singular” (Germano and Waldron, 2006, p. 46). Shou (2026), pp. 82–84) has drawn on this model to develop an analogy between the seed-impression dynamic and the feedback loops of contemporary artificial intelligence systems, observing that the “seed generates manifest activity, manifest activity perfumes seed” structure anticipates the reinforcement learning cycle.^4^

This difference in the temporality of change has practical consequences for how each tradition understands the task of psychological work. In the Jungian model, the work of individuation is a hermeneutic task: the ego encounters symbols from the collective unconscious and interprets them, thereby expanding its self-understanding. Within the span of any individual analysis, the collective unconscious remains effectively constant; what changes is the ego’s awareness of it, while archetypal structures themselves are modified, if at all, only across the long durations of cultural history (Jung, 1969). In the Vijñānavāda model, by contrast, every cognitive act modifies the ālaya-vijñāna that conditions it. The act of observing the mind is not a neutral hermeneutic exercise but a causally efficacious event that alters the very structure being observed. This is the import of the seed-impression theory: cognition is not a mirror reflecting a given psychic landscape but a process that reshapes that landscape with every moment of its operation. The difference between the two traditions is accordingly best stated as a gradient rather than a dichotomy: Jung admits the slow, epochal transformation of archetypal structures, while Vijñānavāda asserts the moment-to-moment modification of the deep mind by every cognitive act. What separates them is the timescale and the mechanism of change, historical sedimentation on one side and the instant-by-instant causal commerce of seeds and manifest activity.

This dynamic architecture carries a theoretical implication that the image of a slowly evolving repository does not capture: the deep layer of mind is not what it is independently of what happens at the surface. Every intentional action deposits its trace in ālaya-vijñāna, modifying the conditions for all subsequent mental events. The deep and surface layers are neither separate nor identical but mutually constitutive. This constitutive reciprocity proves central to understanding what Vijñānavāda can offer at the point where Jung’s framework reaches its limit.

## 4 The illusion of integration: how the collective unconscious mistakes manas’s structural grasping for content awaiting integration

The work of individuation, as Jung describes it, is the progressive integration of unconscious contents into ego-consciousness. This process unfolds through a series of confrontations: with the shadow (repressed personal contents), with the anima or animus (the contrasexual inner figure), and ultimately with the Self (the archetype of psychic totality). The operational logic throughout is integrative: unconscious materials, however threatening or alien they appear, are ultimately to be claimed, recognized, and assimilated by the ego into a more complete self-understanding. Jung insists that this process must remain anchored in the ego from beginning to end: “the encounter with the unconscious has to be seen as the work of one and the same first-personal, individual subject. Indeed, the unconscious cannot be investigated at all without the interaction of the observing consciousness” (Jung, 1958, para. 469). The explicit goal, as Heisig (1999, pp. 90–91) summarizes, is not the dissolution but the reformation of the ego—“not less ego but a reformed ego, less self-sufficient, less centered on controlling perception and experience, more open to unknown and uncontrollable dimensions of mind.”

This integrative model has genuine clinical validity at the level of the personal unconscious. The difficulty arises when the same logic is extended to the collective unconscious. At this point, a question that Jung’s explicit framework does not generate from within itself begins to press: is the impulse toward integration—the drive to make conscious, to claim, to assimilate—itself in need of examination rather than exercise? Vijñānavāda psychology addresses this question through its account of manas, the seventh consciousness.

Manas is defined in the Vijñānavāda system by its characteristic function of “perpetual deliberation” (héng shěn sī liáng, 恒審思量). Manas takes the ālaya-vijñāna as its cognitive object but does not cognize it as it actually is—an impersonal, continuously modified, causally conditioned process. It misapprehends it as a unified, enduring, autonomous self. The Cheng Weishi Lun [Xuanzang (trans.), 2002, juan 4] states: “since it perpetually deliberates, it is called manas. In the pre-transformation stage, it perpetually deliberates the self-image it has grasped; in the post-transformation stage, it perpetually deliberates the reality of no-self.” This perpetual deliberation is accompanied, in the ordinary unawakened state, by four afflictions always co-present: self-delusion (ātma-moha), self-view (ātma-dṛṣṭi), self-conceit (ātma-māna), and self-love (ātma-sneha).^5^

These four afflictions are not merely a cataloguing device. Each maps onto a specific dimension of the integrative logic that structures Jungian individuation. Self-delusion (ātma-moha) corresponds to the fundamental ignorance about the nature of the integrating agent: individuation proceeds without examining whether the “self” doing the integrating is itself a construction rather than a given. Self-view (ātma-dṛṣṭi) corresponds to the persistent assumption that the psyche has a core identity that integration serves to complete—the view that behind the shadow, the anima, the persona, there is a true self waiting to be revealed, a center that integration will finally disclose. Self-conceit (ātma-māna) corresponds to the implicit hierarchy in which the conscious ego is positioned as the evaluator and judge of unconscious contents, the one who decides what to integrate and what remains alien, thus maintaining its sovereignty over the very process that ostensibly relativizes it. Self-love (ātma-sneha) corresponds to the affective attachment to the individuation project itself—the investment in the narrative of self-discovery as inherently valuable and progressive, the pleasure taken in the expanding self as it accumulates deeper layers of integration. In each case, the affliction is not a psychological deficiency that better individuation could remedy; it is a structural feature of the cognitive mode in which individuation operates.

Lin (2000, p. 293) glosses the manas passage with characteristic precision: “the seventh consciousness in the ordinary being’s state perpetually deliberates the eighth consciousness as self with clarity and precision; the seventh consciousness in the Buddha’s state perpetually deliberates the reality of no-self with equal clarity and precision.” What distinguishes these two states is not the presence or absence of deliberation but whether deliberation proceeds from a self-assumption. The self-grasping of manas is not an error correctable by expanding the range of contents admitted to awareness. It is the cognitive form in which awareness operates so long as it is governed by the projective logic of manas.

This diagnosis cuts directly against the Jungian integrative model. The subject who integrates unconscious contents in individuation is, in Vijñānavāda terms, a subject whose entire cognitive activity is structured by the manas-generated assumption of a unitary, enduring cognitive center. Every unconscious content that is “made conscious” is, in the process of being assimilated, absorbed into the economy of this self-assumption. Integration does not dissolve the assumption; it consolidates it. Fù (2011, p. 117) articulates this paradox in terms of the “self-objectification” of mind: erroneous discrimination not only objectifies the presented object but also reflexively objectifies itself as the subject facing that object, so that “erroneous discrimination necessarily manifests as the dyadic structure of grasper and grasped.” The richer the individuated ego becomes, the more elaborately it has organized the field of psychic experience around a center it has never interrogated.

It should be emphasized that this critique does not amount to a dismissal of individuation’s therapeutic achievements. The clinical work of confronting the shadow, integrating dissociated affects, and developing a more differentiated relationship with unconscious contents has genuine psychological value that the Vijñānavāda analysis does not deny. What the analysis identifies is a structural ceiling inherent in the integrative method itself: the ceiling imposed by the self-referential character of the cognitive agent doing the integrating. The four afflictions of manas—self-delusion, self-view, self-conceit, and self-love—are not pathologies to be treated but structural conditions of ordinary cognition that can only be addressed by a transformation of the cognitive mode itself, not by expanding the content that the existing mode processes. This is the sense in which the present article’s critique of individuation is not a negation but an identification of the point at which its own logic calls for resources that its explicit formulation does not supply from within.

## 5 The trap of the self: Jung’s ultimate archetype as a more refined form of manas’s Ātma-Grāha

The critique developed in the previous section targets the methodology of individuation: the integrative logic that governs the process from beginning to end. One might respond that the methodology merely reflects limitations of clinical technique, while the ultimate aim—the Self archetype—represents a genuine transcendence of ego-centered existence. This response, however, does not survive scrutiny unqualified. The present section advances the deeper claim: that the Jungian Self archetype itself, as the theoretical destination of individuation, reproduces at a more sophisticated level the same structural error that manas enacts at the level of lived experience. The progression from Section 4 to Section 5 is therefore not a repetition but an escalation: having shown that individuation’s method perpetuates self-grasping, this section shows that its goal does the same.

The Jungian Self is, in Jung’s own formulation, simultaneously center and totality. In Psychology and Alchemy, Jung (1953, para. 44) writes: “The Self is not only the center, but also the whole circumference which embraces both conscious and unconscious; it is the center of this totality, just as the ego is the center of consciousness.” This formulation deserves to be taken seriously rather than read past. A Self that is circumference as much as center gestures toward a genuine de-centering of the psyche, and any account that treats the Self simply as a consolidated psychic monarch fails to register that gesture. The reading defended in this article is therefore more specific. However radically the Self is extended, in Jung’s texts it remains something given to the psyche as an object of encounter, symbolization, and integration; in Vijñānavāda terms, it retains the structure of an ālambana, a cognitive object correlated with a grasping awareness. The de-centering that the circumference image announces concerns the location and extension of the Self within psychic topography; the transformation at issue in the Vijñānavāda analysis concerns the mode of the cognition for which any center or circumference is given at all. The argument of this section is that the first kind of de-centering, however far it is pushed, does not by itself amount to the second. The Self is simultaneously the telos of individuation and the ground from which it proceeds. Jung associates it with the highest symbolic figures across traditions: “Living in the West, I would have to say Christ instead of ‘Self,’ in the Near East it would be Khidr, in the Far East ātman or Tao or the Buddha, in the Far West maybe a hare or Mandamin, and in cabalism it would be Tifereth” (Jung, 1970, para. 410). The interchangeability of these symbols is for Jung evidence not of cultural relativity but of a universal archetype that each tradition independently expresses. The symbols of divinity and those of the Self are structurally interchangeable: “the symbols of divinity coincide with those of the Self: what, on the one side, appears as a psychological experience signifying psychic wholeness, expresses on the other side the idea of God” (Jung, 1970, para. 339).

Heisig (1999, pp. 92–95) has identified three tacit assumptions sustaining what he calls Jung’s “monarchic psyche.” First, the collective unconscious is conceived as essentially a form of consciousness—“just not an ego-consciousness.” The metaphors Jung employs make this clear: as womb, matrix, or sea, the unconscious is the birthplace of the conscious ego; as storehouse or mine, its contents hold in deposit centuries of experience once conscious to the ego. In either image, the unconscious and consciousness share a single cognitive order. Second, the assumption of a central ego—what Jung once called the “atomic ego” (Jung, 1958, para. 505), a phrase he used to distinguish his position from Eastern nondualism—is extended to the psyche as a whole: just as the ego is the center of consciousness, the Self must be the center of the unconscious, and the individuated Self must be the center of their totality. Third, the tendency of archetypal symbols to seek completion in their opposites crystallizes in symbols of the Self that take the form of a “union of opposites”—a final synthesis in which all structural tensions are harmoniously preserved. The logical form of this synthesis, as Heisig observes, is one of the most primitive forms of abstraction, arguably grounded in basic experiences like the rising and setting of the sun or the interaction of the sexes, and it is no surprise that it appears with such frequency in ritual and myth. What is surprising is that Jung elevates this form to the status of a cosmological principle governing the psyche’s deepest organization without questioning whether the pattern itself might be an artifact of the monarchic structure he has assumed rather than discovered.^6^

Heisig’s critique must itself be qualified, however, in a way that bears directly on the scope of the present argument. As Heisig acknowledges, Jung’s later work moves toward a more apophatic and less centered understanding of the Self: in Mysterium Coniunctionis the language of a psychic monarch recedes before the language of the unus mundus, a unitary ground that is no longer straightforwardly a center possessed by anyone (Jung, 1963). Post-Jungian scholarship has pressed further in this direction. Colman (2008) treats the self not as a pre-given metaphysical entity but as an emergent symbolic construction; Giegerich (2012) dissolves the substantialist reading of the soul altogether in favor of a dialectical one; and Mills (2013) shows that Jung’s professed empiricism coexists with unavoidable metaphysical commitments, so that the question of what the Self finally is cannot be settled by appeal to Jung’s self-description as an empiricist. These developments narrow what this section can responsibly claim. The claim is not that nothing in the Jungian corpus strains against the monarchic structure; the late Jung plainly does. The claim is that the strain remains a strain: the apophatic gestures of the late work are not accompanied by a theoretical account of how cognition could operate otherwise than around a center, and the conceptual machinery of the published model, with the ego-Self axis at its core, is left standing. It is precisely this combination of pressure against a limit and absence of means to cross it that makes the comparison with Vijñānavāda informative, since Vijñānavāda offers exactly such an account in the doctrine of cognitive transformation examined in Section 7.

From a Vijñānavāda perspective, the Self archetype exhibits the characteristic structure of ātma-grāha at a highly refined theoretical level. Manas takes the continuous, impersonal, causally conditioned process of ālaya-vijñāna and converts it into a unified, enduring self. The Self archetype takes the continuous, dynamic, multiply-conditioned processes of the psyche as a whole and converts them into a unified, teleologically organized totality. The structural operation is homologous: a process is converted into an identity, a dynamic is stabilized into a center, and this conversion is experienced not as cognitive distortion but as the recognition of deeper truth. Lusthaus (2002, p. 2) observes that Buddhism consistently employs the language of “grasper and grasped” (grāhaka and grāhya) rather than “subject and object”—a terminological difference that signals a fundamental evaluative asymmetry: where Western metaphysics treats the self-structured character of cognition as a neutral condition of knowledge, Vijñānavāda treats it as a constitutive error.

The structural homology between the Self archetype and manas’s ātma-grāha can be specified more precisely. In both cases, the cognitive operation involves three moves: first, the identification of a continuous process (ālaya-vijñāna or the psyche’s total dynamic); second, the conversion of that process into an entity with the character of permanence, unity, and self-sufficiency; and third, the elevation of that entity to the status of a normative ideal—something to be realized, preserved, or returned to. In the case of manas, these three moves constitute the fundamental error that the entire Vijñānavāda path of practice is designed to undo. In the case of the Self archetype, these three moves constitute the fundamental achievement that the entire process of individuation is designed to accomplish. This symmetry, however, is an interpretive claim rather than a self-evident fact, and it must be defended against an obvious skeptical rejoinder. A Jungian reader can reverse the direction of analysis: if the two traditions describe the same cognitive operation, why not conclude that the Buddhist goal is one more expression of the Self archetype, with the Buddha functioning as a Self symbol alongside Christ, Khidr, and the Tao, exactly as Jung himself proposed? Nothing in the comparison taken by itself rules this reading out, and no neutral standpoint is available from which to adjudicate between it and the Vijñānavāda reading. What the comparison does establish is that each reading is purchased only by presupposing the framework it favors. To interpret samatā-jñāna as an archetypal image is already to apply the Jungian category of the symbol to a doctrine that defines the state in question precisely by the cessation of image-mediated cognition; to interpret the Self archetype as refined ātma-grāha is already to apply the Vijñānavāda category of grasping to a theory that defines the Self as the resolution, not the perpetuation, of psychic conflict. The two frameworks are working with the same cognitive raw material, the mind’s tendency to center its experience, and evaluating it in diametrically opposite ways: what Jung treats as the deepest truth of the psyche, Vijñānavāda treats as the deepest error of cognition. The argument of this article is therefore not that the Vijñānavāda evaluation is demonstrably correct, but that the two evaluations are structurally distinct in a way that assimilationist readings conceal; Section 6 examines what follows once both evaluations are placed under suspicion at once.

Jung himself registered something of this difficulty. He repeatedly insisted that “it must be reckoned a psychic catastrophe when the ego is assimilated by the Self” (Jung, 1959, para. 24), and he described the aim as a reformed ego rather than a dissolved one. Nor is the transcendent function an exception to this pattern. The transcendent function (Jung, 1969) is the closest thing Jung’s model possesses to a mechanism of transformation: the union of conscious and unconscious positions through a mediating symbol, out of which a new attitude emerges. Yet the operation it describes runs through the production and assimilation of symbols, that is, through contents given to an ego that survives the process as its beneficiary. It transforms the ego’s attitude; it does not address the subject-object structure within which attitudes are held. The very formulation of Jung’s caution against assimilation, then, marks the boundary of his explicit model: individuation’s safeguard against catastrophe is the preservation of the ego-center, and the goal is a reformed ego that has recognized its participation in the psychic totality rather than a cognitive mode that no longer requires the ego-center as its organizing principle. The Self archetype, as the endpoint of Jung’s explicit model, appears from the Vijñānavāda standpoint not as the dissolution of self-grasping but as its most elaborate expression: a self that has incorporated the shadow, negotiated the contrasexual interior, and recognized its participation in the totality of the psyche—and that understands this recognition as the completion of a journey that began, continued, and ended in the first-person singular.

## 6 The meditation cushion and the consulting room: the epistemological boundary neither can cross

The preceding argument has moved asymmetrically in Vijñānavāda’s favor. The present section addresses the reciprocal constraint. Both traditions are bounded by the epistemological conditions of their respective methods, and each can illuminate what the other cannot precisely because of the different vantage points their methods supply.

### 6.1 The epistemological boundary on each side

Jung’s method is anchored in the consulting room: in the clinical encounter, in the analysis of dreams and active imagination, in the comparative study of symbolic and mythological material. This method’s strength is its capacity to address the question of meaning at the level at which individuals actually encounter it—embedded in personal history, cultural context, and the texture of symbolic experience. Its structural constraint is the one Jung acknowledged: “the unconscious cannot be investigated at all without the interaction of the observing consciousness” (Jung, 1958, para. 469). Every insight depth psychology achieves about the unconscious is an insight achieved by the ego, expressed in symbolic language the ego can receive, and organized around the questions the ego is capable of formulating. This is not a contingent limitation but a structural feature. The ego’s capacity for self-investigation cannot encompass the conditions of its own possibility, because those conditions are precisely what the ego does not directly access. Jungian depth psychology is a hermeneutics of the ego’s encounter with what lies beyond its ordinary horizon—but it remains a hermeneutics whose final interpretive authority belongs to the ego. It can approach the limit of ego-organized cognition; it cannot step outside that organization to examine it.^7^

This structural constraint means that Jung cannot access the cognitive mode that Vijñānavāda’s most significant claims presuppose. In the Vijñānavāda system, knowledge of ālaya-vijñāna and the operation of manas is not primarily propositional knowledge acquired through reflection on symbolic materials. As Nī (2002, p. 65) observes, “the understanding of ālaya-vijñāna is determined by the level of contemplative attainment reached through practice; for those who have not reached the corresponding level, one can only stop here and set the question aside.” Husserl encountered an analogous limit when attempting to characterize “ultimate consciousness” (letztes Bewußtsein): he acknowledged that the means of accessing this level of mind was “less intuition than inference” (Husserl, 1966, p. 382), and twice declined to “descend into the obscure depths of the ultimate consciousness constituting the temporality of all experience” (Husserl, 1973, §81, §85). The Cheng Weishi Lun [Xuanzang (trans.), 2002, juan 2] makes a structurally parallel acknowledgment: “since the nature of this eighth consciousness is extremely subtle, it is demonstrated through its functions and activities”—ālaya-vijñāna cannot be directly grasped but only inferred.^8^

But Vijñānavāda faces a reciprocal limitation. Its analysis of mind was developed within a soteriological horizon oriented toward identifying and eliminating the cognitive conditions for suffering and cyclic existence. This orientation produces extraordinary precision in the analysis of the internal structure of cognition but is associated with an underdevelopment of the dimensions that Jung’s psychology addresses most richly: the symbolic and cultural embeddedness of psychic experience, the therapeutic value of the encounter between ego and unconscious, and the significance of individuation as a historically situated human achievement. Segall (2023) has observed that contemporary practitioners integrating Buddhist approaches with Western psychotherapy consistently “naturalize” Buddhist practice—bracketing its soteriological commitments and reframing the goal as psychological flourishing within a single life—a move reflecting a genuine theoretical need that classical Vijñānavāda does not meet. Fù (2013) has argued that Buddhist epistemological traditions, from Sarvāstivāda to Sautrāntika and into the Yogācāra synthesis, were shaped by distinctly soteriological motivations that cannot be extracted without loss. Nī (2019) has made the further observation that the Western concept of “consciousness” corresponds not to any single Buddhist vijñāna but to the entire field designated by the compound citta-manas-vijñāna (mind-thought-consciousness)—a scope difference that has practical consequences for how comparison is conducted.

The epistemological impasse thus runs symmetrically. Jung cannot access the meditative mode of cognition that Vijñānavāda’s most important claims presuppose; Vijñānavāda cannot generate, from within its own resources, an account of the culturally embedded, relationally constituted, and symbolically mediated dimensions of mind that Jung’s consulting-room method uniquely illuminates. This mutual limitation is not a defect of either tradition but a clarification of the theoretical space each occupies—and of what a genuinely integrative account of mind would require.

What would such an integrative account look like in practice? The question exceeds the scope of this article, but two observations may be offered. First, any account that seeks to combine Jungian and Vijñānavāda perspectives would need to respect the irreducible difference in their epistemic methods: the hermeneutic-clinical method of depth psychology and the contemplative-experiential method of Buddhist practice cannot be collapsed into a single procedure without distorting both. What can be attempted is a sequential relationship, in which the achievements of one method serve as the precondition for the other—a point to which the final section will return. Second, such an account would need to address the translation problem that Nī (2019) has identified: the Western concept of “consciousness” does not map cleanly onto any single Buddhist category, and the temptation to treat ālaya-vijñāna as simply “the Buddhist unconscious” or manas as “the Buddhist ego” must be resisted if the comparison is to yield genuine theoretical insight rather than superficial analogy.

### 6.2 The reflexive turn: applying the hermeneutics of suspicion to Vijñānavāda itself

A further limitation must be confronted, one that the argument of this article incurs by its own procedure. The preceding sections have used Vijñānavāda’s account of mind as the analytical instrument and Jung’s account as the object of analysis; the instrument itself has so far escaped examination. Consistency requires that the hermeneutics of suspicion applied to the Self archetype be applied with equal force to the eight-consciousness model. Is that model a description of cognitive architecture, or is it a culturally conditioned symbolic system, the product of Indian śāstric debate, monastic contemplative culture, and a soteriological project whose categories were forged to serve liberation rather than description? The historical scholarship already cited points firmly toward conditioning: Schmithausen (1987) reconstructs the ālaya-vijñāna as a solution to doctrinal problems internal to a specific textual tradition, and Fù (2013) shows that Buddhist epistemology from Sarvāstivāda through the Yogācāra synthesis was shaped throughout by soteriological motivations that cannot be subtracted without remainder. The eight-consciousness model did not fall from the sky; it was built, layer by layer, under determinate cultural and religious pressures.

Jung himself supplies the sharpest form of this objection. In his psychological commentary on The Tibetan Book of the Dead, he warns explicitly against taking Eastern maps of mind as literal descriptions, insisting that such materials become available to Western understanding only through psychological translation, as symbolic expressions of the collective unconscious rather than as veridical cartography (Jung, 1958, paras. 831–858). Applied to the present case, the Jungian rejoinder is straightforward: the eight consciousnesses, the seeds, the four wisdoms, and the figure of the Buddha are archetypal images, and the doctrine of turning consciousness into wisdom is a symbolic representation of individuation itself, projected onto a cosmological canvas. On this reading, the present article has not diagnosed a limit of Jung’s framework from a vantage point beyond it; it has merely redescribed one symbolic system in the vocabulary of another.

The disagreement this objection opens is genuinely meta-level, and it cannot be resolved by either party without circularity, since each framework possesses the resources to redescribe the other. What can be said is that Vijñānavāda is not naive on the point. The tradition carries its own deconstructive devices. The Cheng Weishi Lun opens by declaring that self and dharmas alike are established by provisional designation [Xuanzang (trans.), 2002, juan 1], a statement that places the system’s own categories under the sign of the provisional from its first sentence. The three-natures doctrine extends this self-relativization: every conceptual scheme, the Buddhist scheme included, operates at the level of imaginative construction (parikalpita) so long as it is taken as a description of independently existing entities. The tradition’s self-understanding, in other words, already concedes that the eight-consciousness model is a raft and not a riverbed. Contemporary analytic philosophy of Buddhism makes a convergent demand from outside the tradition: Albahari (2014) argues that the epistemic credentials of no-self claims must be established by argument rather than assumed on doctrinal authority, which is precisely the standard to which the present comparison should be held.

Acknowledging this double suspicion changes what the article’s conclusion can claim. If both frameworks are culturally conditioned symbolic systems, the comparison cannot establish that Vijñānavāda describes a real cognitive transformation lying beyond a real Jungian limit. What it can establish is a structural difference between the two systems as theories: one organizes its account of mind around an ineliminable center and provides no account of cognition without one; the other treats the center as a revisable interpretive imposition and provides a detailed, internally coherent account of cognitive activity proceeding without it, together with an account of its own categories as provisional. Whether that second account answers to anything actual is a question this article cannot settle and does not claim to settle. The conclusion defended in Section 7 should be read under this proviso throughout.

## 7 Turning consciousness into wisdom: Vijñānavāda’s theory of cognitive transformation as the unfinished path of Jungian individuation

The asymmetry between the two traditions, when properly understood, is not a basis for declaring one superior. It is a specification of what each tradition offers the other at the point where the other’s resources run out. This final section develops the most important instance of that complementarity: the Vijñānavāda doctrine of “turning consciousness into wisdom” as a theoretical continuation of what Jungian individuation initiates but does not, on its explicit terms, complete.

The doctrine of zhuǎn shí chéng zhì (轉識成智) describes a transformation in which each of the eight consciousnesses undergoes a fundamental change in mode of operation. The first five sense-consciousnesses are transformed into “wisdom of accomplished action” (kṛtyānuṣṭhāna-jñāna); the sixth mental consciousness into “wisdom of wondrous discernment” (pratyavekṣaṇā-jñāna); manas into “wisdom of equality” (samatā-jñāna); and ālaya-vijñāna into “great mirror wisdom” (mahādarśa-jñāna). The Cheng Weishi Lun [Xuanzang (trans.), 2002, juan 10] specifies that after transformation, the eighth consciousness may still be designated “consciousness”—now pure or undefiled consciousness (amala-vijñāna)—“because it still takes all phenomena as its cognitive object,” but what that designation refers to is, in substance, wisdom rather than defiled cognition.^9^ This precision is philosophically decisive: what changes is not the presence of cognitive activity but the structural orientation from which that activity proceeds. The eight consciousnesses continue to function; what ceases is the self-grasping that governed their functioning.

The transformation of manas into samatā-jñāna is the hinge on which this doctrine turns in relation to the Jungian problematic. The Cheng Weishi Lun [Xuanzang (trans.), 2002, juan 4] states: “in the pre-transformation stage, manas perpetually deliberates the self-image it has grasped; in the post-transformation stage, manas perpetually deliberates the reality of no-self.” The function of perpetual deliberation does not disappear; what changes is whether it proceeds from a self-assumption. The transformation of manas is not the elimination of deliberative cognitive activity; it is the removal of the compulsion to organize every cognitive event around the fiction of a stable self-center. What remains after transformation is not a cognitive void but a cognitive activity freed from its most fundamental misorientation.

The notion of a transformation of cognitive mode need not remain confined to soteriological vocabulary; recent psychological research supplies constructs that allow it to be stated, and in principle investigated, in the idiom of empirical psychology. Decentering, the capacity to relate to thoughts and feelings as passing mental events rather than as deliverances of a substantial self, has been given a detailed metacognitive process model (Bernstein et al., 2015) and marks a first, content-level approximation. The taxonomy of Dahl et al. (2015) goes further, distinguishing a deconstructive family of meditation practices whose specific target is self-related processing itself rather than attention or affect, which corresponds closely to the level at which the transformation of manas is defined. Vago and Silbersweig (2012) frame self-transcendence as a decoupling of experience from self-referential processing, and Josipovic (2014) identifies nondual awareness, a mode of consciousness in which the subject-object structure of experience is attenuated or absent, as a distinct and empirically tractable construct; reports of selfless states have begun to receive systematic treatment (Millière et al., 2018), and the trajectory from self to nonself has been proposed as a psychological theory in its own right (Shiah, 2016). None of these constructs is equivalent to āśraya-parāvṛtti, the transformation of the basis, which is defined within a soteriological framework that these research programs deliberately bracket. They suffice, however, to show that cognitive mode transformation designates a candidate psychological phenomenon, specifiable as a change in the structural orientation of cognition rather than in its contents, and not merely a doctrinal postulate.

This distinction maps precisely onto a question that Jung’s explicit model does not formulate. Jung’s process accomplishes—through integration of shadow, anima/animus, and encounter with the Self—a genuine transformation of the ego from a narrow, self-enclosed locus of consciousness to a more porous and symbolically enriched subject of experience. What individuation, as Jung explicitly formulated it, does not accomplish, and what his theoretical framework does not formulate as a goal, is the transformation of the cognitive mode itself, from the mode governed by manas’s self-assumption to the mode that proceeds without it. The distinction between transforming the contents of ego-consciousness and transforming the structural orientation of consciousness as such is the precise point at which Vijñānavāda theory offers a continuation at the point where Jung’s explicit theory stops. Ellenberger (1970) documented the discovery of the unconscious as one of the decisive achievements of modern Western thought; what his account also makes visible, in retrospect, is the structural ceiling this discovery implies.

Jung approached this limit in late life. In the German original of his memoir, he speaks of “an extension of the ego or consciousness achieved in old age” (Jung, 1961/1962, p. 229); the English translation omits the reference to the ego (Heisig, 1999, p. 91, n. 27), producing a sentence that suggests development beyond ego-involvement. The German is more honest: even at the furthest reach of individuation as Jung experienced it, the extension remained an extension of the ego rather than a transformation of the mode in which the ego operates. The Vijñānavāda concept of samatā-jñāna names exactly what would have to change for the extension Jung describes to become the transformation his explicit framework did not formulate.

Two further Vijñānavāda concepts sharpen this specification. The first is the tripartite account of cognition: the division of each cognitive act into subjective aspect (darśana-bhāga), objective aspect (nimitta-bhāga), and self-witnessing aspect (svasaṃvitti-bhāga). Nī (2008, pp. 16–26) has argued that the self-witnessing division constitutes a complete cognitive act in its own right: consciousness can authenticate its own activity without the mediation of a posited self-center. In the unawakened state, the self-witnessing function is governed by manas: every moment of cognitive self-awareness is experienced as the awareness of an “I” engaged in knowing. After the transformation of manas, the self-witnessing function can operate without this projection: cognitive events can be witnessed without being attributed to a witnessing self. This is not the elimination of awareness but its structural reconfiguration—a form of awareness that is self-aware without being self-projecting. The parallel with individuation: Jung’s reformed ego is a subject that has learned to witness the operations of the unconscious without being overwhelmed; what the transformation of manas achieves is a witnessing that no longer requires a subject in Jung’s sense.^10^

The second concept is the theory of seeds and impressions as dynamic karmic inheritance. Waldron (2003b, pp. 145–191) has argued that ālaya-vijñāna is “neither an agent nor a faculty, much less an ātman in disguise,” but a conceptual rubric for continuous subliminal processes—bodily awareness, subliminal perception, the implicit influences of language and habit—not organized by a self-center yet constituting the ground of all subsequent experience. When these processes are no longer misinterpreted by manas as constituting a self, their actual character becomes available: not a center but a field, not a subject but a process, not a destination but an ongoing and open-ended becoming. Sun and Feng (2020, pp. 1014–1015) have described how the eight-consciousness model distributes cognitive functions across multiple layers—from the “display layer” of the six sense-consciousnesses to the “system layer” of manas and the “warehouse layer” of ālaya-vijñāna’s seed-store—a structural analysis that reinforces the point that no single layer constitutes a “self” in the Jungian monarchic sense.

What this architectural analysis reveals is that the Vijñānavāda model of mind does not locate selfhood in any single consciousness but treats it as an artifact of manas’s interpretive activity—a projection imposed upon a multi-layered, multi-directional process that is, in itself, centerless. The Jungian Self archetype, by contrast, locates selfhood at the apex of the psyche’s organization, as the principle that unifies all the layers into a centered totality. The difference is not merely terminological. It reflects two fundamentally different accounts of what it means for mind to be organized: one in which organization requires a center, and one in which organization can proceed without one. The Vijñānavāda claim is not that centerless organization is empirically demonstrated—it is, after all, the result of a contemplative practice that most people have not undertaken—but that it is theoretically coherent and practically achievable. This claim is what makes Vijñānavāda a possible continuation of Jung rather than a mere alternative: it takes the question Jung raised—can the ego be transformed?—and answers it with a specificity that Jung’s own framework could not provide.

A final observation concerns the relationship between theory and practice. The Vijñānavāda doctrine of cognitive transformation is not merely a theoretical proposition but the description of a practical path—a path that involves specific meditative and ethical disciplines, sustained over extended periods, and guided by a tradition of transmission. The theoretical analysis offered in this article cannot substitute for that practice, any more than a description of clinical technique can substitute for the experience of analysis. What the theoretical analysis can do is specify the structural relationship between the two traditions with sufficient precision to clarify what each has to offer the other. Jung’s depth psychology provides the indispensable therapeutic and hermeneutic work of confronting the personal and collective unconscious—work that, in the Vijñānavāda view, would correspond to the preliminary purification of gross afflictions that precedes the subtler work of transforming the cognitive mode itself. Vijñānavāda provides the conceptual framework for understanding what that subtler work involves and what it would mean to complete it. Neither tradition alone addresses the full scope of the problem. Together, they trace a path from the surface of consciousness to the structural conditions that make consciousness possible, and from there to the possibility of a cognitive mode that is no longer organized around the fiction of a permanent self.

The relationship between Jung’s individuation and Vijñānavāda’s cognitive transformation is one not of competition but of sequence. Jung’s individuation addresses the content of unconscious life—what has been split off, repressed, or denied in the course of personal and collective history—and its clinical and cultural achievements are not diminished by the analysis offered here. Vijñānavāda addresses the form of cognitive activity itself—the structural orientation from which integration proceeds, and whether that orientation can itself be changed. Where Jung brings the ego to the edge of a territory his model does not enter, Vijñānavāda offers a map of what may lie beyond, a map whose own epistemic status was examined in Section 6. Individuation may then be read as a precondition for what Vijñānavāda describes, and Vijñānavāda theory as a possible continuation of what individuation approaches. On this reading, the Self is not the destination of the mind’s journey but the last landmark before the road changes. The reading is offered as a structural hypothesis for further comparative and empirical work, not as a settled verdict on either tradition.

## Data Availability

The original contributions presented in the study are included in the article/supplementary material, further inquiries can be directed to the corresponding author.

## Footnotes

1 Heisig’s examination covers Jung’s engagement with Zen, Tibetan Buddhism, Taoism, and Yoga, showing in each case how Jung’s interpretive frame assimilates rather than engages. Clarke (1994) offers a more sympathetic account but reaches a convergent conclusion regarding the theoretical limits of Jung’s Eastern engagement.

2 The methodological informality Heisig identifies corresponds to a broader hermeneutic orientation in Jung’s work: its characteristic movement is from clinical observation to mythological comparison to theoretical generalization, with each stage understood as converging confirmation of the core hypotheses.

3 The term ālaya-vijñāna combines ālaya (Sanskrit: dwelling, abode, repository) with vijñāna (consciousness, discernment). The Chinese rendering cáng shí (藏識, “store-consciousness”) captures the threefold sense of the Cheng Weishi Lun: the capacity to store (néng cáng, 能藏), the status of being stored (suǒ cáng, 所藏), and the characteristic of being grasped-as-self (zhí cáng, 執藏).

4 All quotations from the Cheng Weishi Lun are translated from the Chinese text (CBETA T31, no. 1585) by the author, with reference to Cook (1999) and Lin (2000) for interpretive guidance. Jung CW quotations follow standard volume:paragraph notation.

5 Manas (末那識) should be distinguished from the sixth consciousness, mano-vijñāna (意識). The seventh consciousness is transliterated as “manas” in Chinese Buddhist usage; the sixth is translated as “consciousness” (yìshí). The distinction is between the structural self-grasping function (manas) and the discerning cognitive function (mano-vijñāna) that depends on it.

6 Heisig (1999, p. 94) notes that the monarchic psyche and Christian monotheism “support the same tacit assumption.” The implication is that the Self archetype may be not a universal discovery but a culturally specific projection of the monotheistic God-image onto the structure of the psyche.

7 Jung’s late reflections on the Kundalini Yoga are instructive here. He observed that the Eastern unconscious is “above” while the Western is “below” (Jung, 1975, pp. 12–13), implying that the psychological starting points are so different that direct comparison of methods may be misleading.

8 The epistemological situation—where the deepest layer of mind can only be inferred from its effects—is shared by Vijñānavāda, Husserlian phenomenology, and Jungian depth psychology, despite their substantial differences. Nī (2002, pp. 65–70) develops this parallel in detail.

9 The status of consciousness after transformation is debated within the Vijñānavāda tradition. Paramārtha (6th century) distinguished a ninth consciousness, amala-vijñāna, as a separate level. Xuanzang’s Cheng Weishi Lun rejects this distinction. For the debate, see Yinshun (1992, pp. 144–160).

10 The distinction between abolishing subjectivity and reconfiguring it is critical for avoiding the misreading, common in Western presentations, that Buddhist “no-self” (anātman) amounts to a denial of experience. The transformation of manas does not eliminate the cognitive agent; it reconstitutes cognitive activity on a basis that no longer requires the fiction of a unitary center.

## References

[ref1] Albahari M. (2014). Insight knowledge of no self in Buddhism: an epistemic analysis. Philos. Imprint 14, 1–30. Available online at: http://hdl.handle.net/2027/spo.3521354.0014.021

[ref2] Alexander F. (1931). Buddhistic training as an artificial catatonia (the biological meaning of psychic occurrences). Psychoanal. Rev. 18, 129–145.

[ref3] Bernstein A. Hadash Y. Lichtash Y. Tanay G. Shepherd K. Fresco D. M. (2015). Decentering and related constructs: a critical review and metacognitive processes model. Perspect. Psychol. Sci. 10, 599–617. doi: 10.1177/1745691615594577, 26385999 PMC5103165

[ref4] Clarke J. J. (1994). Jung and Eastern Thought: A Dialogue with the Orient. London: Routledge.

[ref5] Colman W. (2008). On being, knowing and having a self. J. Anal. Psychol. 53, 351–366. doi: 10.1111/j.1468-5922.2008.00731.x, 18494672

[ref9001] Cook F. H. (1999). Three Texts on Consciousness Only. Berkeley, CA: Numata Center for Buddhist Translation and Research.,

[ref6] Coward H. (1985). Jung and Eastern Thought. Albany, NY: SUNY Press.

[ref7] Dahl C. J. Lutz A. Davidson R. J. (2015). Reconstructing and deconstructing the self: cognitive mechanisms in meditation practice. Trends Cogn. Sci. 19, 515–523. doi: 10.1016/j.tics.2015.07.001, 26231761 PMC4595910

[ref8] Ellenberger H. F. (1970). The Discovery of the Unconscious: The History and Evolution of Dynamic Psychiatry. London: Allan Lane/Penguin Press.

[ref9] Freud S. (1927/1961). The Future of an Illusion (J. Strachey, Trans.). New York: Norton.

[ref10] Fromm E. Suzuki D. T. de Martino R. (1960). Zen Buddhism and Psychoanalysis. New York: Harper and Brothers.

[ref11] Fù X. (2011). What is “vijñapti-mātra”? On the positioning of vijñapti from a phenomenological perspective. Zhongguo Zhexueshi 3, 114–120.

[ref12] Fù X. (2013). How is cognition possible? Buddhist epistemology from Sarvāstivāda to Sautrāntika. Nanjing Daxue Xuebao 50, 84–96.

[ref13] Germano D. F. Waldron W. S. (2006). “A comparison of ālaya-vijñāna in Yogācāra and Dzogchen,” in Buddhist Thought and Applied Psychological Research, eds. Nauriyal D. K. Drummond M. S. Lal Y. B. (London: Routledge), 36–68.

[ref14] Giegerich W. (2012). What Is Soul? New Orleans: Spring Journal Books.

[ref15] Heisig J. W. (1999). Jung, Christianity and Buddhism. Nanzan Bulletin 23, 75–105.

[ref16] Husserl E. (1966). Zur Phänomenologie des inneren Zeitbewußtseins (1893–1917). Husserliana X. The Hague: Martinus Nijhoff.

[ref17] Husserl E. (1973). Ideen zu einer reinen Phänomenologie und phänomenologischen Philosophie, Bd. I. Husserliana III/1. The Hague: Martinus Nijhoff.

[ref18] Josipovic Z. (2014). Neural correlates of nondual awareness in meditation. Ann. N. Y. Acad. Sci. 1307, 9–18. doi: 10.1111/nyas.12261, 24033505

[ref19] Jung C. G. (1953). Psychology and Alchemy. Collected Works, Vol. 12. Princeton: Princeton University Press.

[ref20] Jung C. G. (1958). Psychology and Religion: West and East. Collected Works, Vol. 11. Princeton: Princeton University Press.

[ref21] Jung C. G. (1959). Aion: Researches into the Phenomenology of the Self. Collected Works, Vol. 9/2. Princeton: Princeton University Press.

[ref22] Jung C. G. (1961/1962). Erinnerungen, Träume, Gedanken [Memories, Dreams, Reflections]. Buchclub Ex Libris (German, 1961); New York: Pantheon (English, 1962).

[ref23] Jung C. G. (1963). Mysterium Coniunctionis. Collected Works, Vol. 14. Princeton: Princeton University Press.

[ref24] Jung C. G. (1968). The Archetypes and the Collective Unconscious. Collected Works, Vol. 9/1. Princeton: Princeton University Press.

[ref25] Jung C. G. (1969). The Structure and Dynamics of the Psyche. Collected Works, Vol. 8. Princeton: Princeton University Press.

[ref26] Jung C. G. (1970). Civilization in Transition. Collected Works, Vol. 10. Princeton: Princeton University Press.

[ref27] Jung C. G. (1975). Psychological commentary on the kundalini yoga: part 1. Spring, 1–32.

[ref28] Lin G. (2000). Cheng Weishi Lun Zhijie. [Direct Interpretation of the Demonstration of Consciousness Only]. Fudan University Press.

[ref29] Lusthaus D. (2002). Buddhist Phenomenology: A Philosophical Investigation of Yogācāra Buddhism and the Ch’eng Wei-shih Lun. Curzon: Routledge.

[ref30] Millière R. Carhart-Harris R. L. Roseman L. Trautwein F.-M. Berkovich-Ohana A. (2018). Psychedelics, meditation, and self-consciousness. Front. Psychol. 9:1475. doi: 10.3389/fpsyg.2018.01475, 30245648 PMC6137697

[ref31] Mills J. (2013). Jung’s metaphysics. Int. J. Jungian Stud. 5, 19–43. doi: 10.1080/19409052.2012.671182

[ref32] Moacanin R. (1986). Jung’s Psychology and Tibetan Buddhism: Western and Eastern Paths to the Heart. Wisdom Publications.

[ref33] Nī L. (2002). On self-consciousness and ego-consciousness in Vijñānavāda and phenomenology. Zhongguo Xueshu 3, 60–90.

[ref34] Nī L. (2008). The basic meaning of svasaṃvitti-bhāga in Vijñānavāda. Xueshu Yanjiu 1, 16–26.

[ref35] Nī L. (2019). Consciousness as a philosophical problem and a scientific topic. Ziran Bianzhengfa Tongxun 41, 31–41. doi: 10.15994/j.1000-0763.2019.10.005

[ref36] Roesler C. (2012). Are archetypes transmitted more by culture than biology? Questions arising from conceptualizations of the archetype. J. Anal. Psychol. 57, 223–246. doi: 10.1111/j.1468-5922.2011.01963.x, 22444357

[ref37] Schmithausen L. (1987). Ālayavijñāna: On the Origin and Early Development of a Central Concept of Yogācāra Philosophy. Tokyo: International Institute for Buddhist Studies.

[ref38] Segall S. Z. (2020). Buddhism and Human Flourishing: A Modern Western Perspective. London: Palgrave Macmillan.

[ref39] Segall S. Z. (2023). Buddhism and Western Psychology. Oxford Research Encyclopedias: Religion. Oxford: Oxford University Press.

[ref40] Shiah Y.-J. (2016). From self to nonself: the nonself theory. Front. Psychol. 7:124. doi: 10.3389/fpsyg.2016.00124, 26869984 PMC4740732

[ref41] Shou B. (2026). An AI Vijnanavada-based agent-agency-action framework. Shanghai Shifan Daxue Xuebao Zhexue Shehui Kexue Ban 1, 76–92.

[ref42] Sun C. Feng C. (2020). A five-level hierarchy of the subject with mind based on the five-aggregate model in Buddhism. Psychol. Sci. 43, 1007–1016. doi: 10.16719/j.cnki.1671-6981.20180437

[ref43] Vago D. R. Silbersweig D. A. (2012). Self-awareness, self-regulation, and self-transcendence (S-ART): a framework for understanding the neurobiological mechanisms of mindfulness. Front. Hum. Neurosci. 6:296. doi: 10.3389/fnhum.2012.00296, 23112770 PMC3480633

[ref44] Waldron W. S. (2003a). The Buddhist Unconscious: The Ālaya-vijñāna in the Context of Indian Buddhist Thought. Curzon: Routledge.

[ref45] Waldron W. S. (2003b). “Common ground, common cause: Buddhism and science on the afflictions of identity,” in Buddhism and Science: Breaking New Ground, ed. Wallace B. A. (New York: Columbia University Press), 145–191.

[ref46] Xiong S. (1985). Fojia Mingxiang Tongshi. [Comprehensive Explanation of Buddhist Technical Terms]. Beijing: Zhongguo Da Baike Quanshu Press.

[ref47] Xuanzang (trans.) (2002). Cheng Weishi Lun. [Demonstration of Consciousness Only]. CBETA Digital Edition (T31, no. 1585. Original 7th century CE; English trans. in F. H. Cook, Three Texts on Consciousness Only, Numata Center, 1999.) Taipei: CBETA Digital Edition.

[ref48] Yinshun (1992). Weishi Xue Tanyuan. [Exploring the Sources of Vijñānavāda]. Taipei: Zhengwen Press.

